# Factors Associated with Gestational Week of Diagnosis Among Pregnancies with Prenatally Recognized Congenital Heart Disease: A Tertiary Referral-Centre Cohort Study

**DOI:** 10.3390/children13081090

**Published:** 2026-08-17

**Authors:** Olivia Lili Szepesi, Virág Bartek, Gréta Kiss, Réka Hodula, István Szabó, Artúr Beke

**Affiliations:** Department of Obstetrics and Gynecology, Semmelweis University, 1082 Budapest, Hungary; szepesi.olivia@stud.semmelweis.hu (O.L.S.); bartek.virag@stud.semmelweis.hu (V.B.); kiss.greta@stud.semmelweis.hu (G.K.); hodula.reka@stud.semmelweis.hu (R.H.); szabo.istvan@semmelweis.hu (I.S.)

**Keywords:** fetal echocardiography, congenital heart defects, prenatal diagnosis, gestational age, maternal age, down syndrome, risk factors

## Abstract

**Highlights:**

**What are the main findings?**

- Univariable analyses identified several maternal, fetal, chromosomal, and lesion-specific factors associated with the gestational week of prenatal diagnosis, including chromosomal abnormalities, Down syndrome, oligohydramnios, and selected cardiac diagnoses.
- In the revised multivariable model, increasing maternal age and later calendar year were associated with earlier prenatal diagnosis, whereas oligohydramnios, coarctation of the aorta/aortic arch stenosis, and pulmonary stenosis were associated with later diagnosis. Down syndrome showed only a borderline association with earlier diagnosis.

**What are the implications of the main findings?**

- The contrast between univariable and multivariable findings suggests that apparent differences in diagnostic timing may partly reflect correlated maternal, fetal, temporal, chromosomal, and lesion-specific characteristics.
- The gestational week of prenatal CHD diagnosis appears to be shaped by surveillance pathways, temporal changes in prenatal care, fetal imaging conditions, and lesion-specific sonographic visibility rather than by individual risk factors alone.

**Abstract:**

**Background**: Prenatal diagnosis of congenital heart defects (CHD) is important for perinatal planning, yet the gestational week at which CHD is diagnosed varies considerably. In referred cohorts, diagnostic timing may be influenced by maternal, fetal, temporal, and lesion-specific factors. This study examined factors associated with the gestational week of prenatal diagnosis among pregnancies with prenatally recognized and subsequently confirmed CHD referred to a national tertiary fetal cardiology centre. **Methods**: This study analyzed 833 pregnancies with confirmed CHD managed between 2005 and 2020. The primary outcome was the gestational week of prenatal diagnosis. Univariable associations with binary predictors were assessed using Student’s *t*-test, Welch’s *t*-test, or the Mann–Whitney U test according to distributional assumptions. A primary fully adjusted multivariable linear regression model with heteroscedasticity-robust standard errors was used to evaluate variables associated with the gestational week of diagnosis. The model included maternal age, calendar year as a continuous variable, twin pregnancy, amniotic fluid abnormalities, chromosomal status, and selected lesion-specific diagnoses. Chromosomal status was modelled hierarchically as no chromosomal abnormality, Down syndrome, or other chromosomal abnormality. **Results**: In univariable analyses, advanced maternal age, Down syndrome, and chromosomal abnormalities were associated with earlier diagnosis, whereas oligohydramnios was associated with later diagnosis. In the primary fully adjusted multivariable model, increasing maternal age was associated with earlier diagnosis (β = −0.169 weeks per year, 95% CI −0.238 to −0.099; *p* < 0.001), as was later calendar year (β = −0.211 weeks per year, 95% CI −0.311 to −0.111; *p* < 0.001). Coarctation of the aorta/aortic arch stenosis (β = 2.276 weeks, 95% CI 0.787 to 3.764; *p* = 0.003), pulmonary stenosis (β = 1.996 weeks, 95% CI 0.376 to 3.617; *p* = 0.016), and oligohydramnios (β = 2.097 weeks, 95% CI 0.228 to 3.965; *p* = 0.028) were associated with later prenatal diagnosis. Down syndrome showed a borderline association with earlier diagnosis (β = −1.350 weeks, 95% CI −2.702 to 0.002; *p* = 0.050). The model explained 12.2% of the variability in the gestational week of diagnosis, with an adjusted R^2^ of 0.107. **Conclusions**: In this tertiary referral-centre cohort of pregnancies with prenatally recognized and confirmed CHD, the gestational week of prenatal diagnosis was associated with maternal age, calendar year, oligohydramnios, and selected lesion-specific diagnoses. These findings should be interpreted as observational associations within an already recognized referral cohort rather than determinants of prenatal CHD detection in the general pregnant population. Future studies should incorporate referral pathways, screening history, ultrasound quality, operator experience, fetal position, and lesion-specific imaging characteristics to better characterize factors influencing the timing of prenatal CHD diagnosis.

## 1. Introduction

Congenital heart defects (CHDs) represent the most common birth defect, affecting nearly 1% of live births and constituting a leading cause of infant morbidity and mortality [1]. Prenatal detection of CHD is critical for optimizing perinatal outcomes, as it enables timely counselling, focused diagnostic testing, and optimal delivery planning [1,2]. Prenatal diagnosis has been shown to reduce neonatal morbidity and mortality by allowing clinicians to optimize prenatal care, prevent hemodynamic compromise, and ensure appropriate postnatal stabilization [3]. Specifically, infants diagnosed prenatally demonstrate improved arterial pH, better oxygenation, less myocardial dysfunction, and reduced end-organ injury compared to those diagnosed postnatally [3,4].

Despite advances in fetal cardiac screening, the gestational age at CHD diagnosis varies considerably, ranging from first-trimester detection to postnatal diagnosis [5,6]. Multiple factors have been associated with recognition timing, including maternal characteristics, fetal factors, and the complexity of the cardiac lesion [7,8]. Advanced maternal age has been linked to both increased CHD prevalence and earlier prenatal detection, while chromosomal abnormalities, particularly trisomy 21 and other aneuploidies, are present in 30–40% of fetuses with CHD and may influence detection patterns [3,5,7,9]. Additionally, extracardiac abnormalities, including amniotic fluid volume discrepancies, have been associated with CHD and may affect diagnostic timing [10,11].

However, the independent contributions of these factors to recognition timing remain poorly characterized. Most previous studies have examined individual risk factors in isolation using univariable analyses, which cannot account for potential confounding or correlation among variables [7]. A multivariable analytical approach is essential to determine which factors exert adjusted association on recognition timing and to what extent observed associations reflect true causal relationships versus confounding by other variables.

The objective of this study was to investigate the relationship between maternal and fetal parameters, including maternal age, twin pregnancy, amniotic fluid volume abnormalities, chromosomal abnormalities, CHD severity, and the gestational age at CHD diagnosis. We hypothesized that while multiple factors would demonstrate significant associations in univariable analyses, only a subset would retain independent predictive value in multivariable modelling. Understanding these independent variables supports clinical awareness, and improves understanding of diagnostic timing.

## 2. Materials and Methods

### 2.1. Study Design and Setting

This analysis represents a component of an ongoing prospective cohort study conducted at the Department of Obstetrics and Gynecology, Semmelweis University, Budapest, Hungary, the national tertiary referral centre for fetal congenital anomalies. The study included pregnancies managed between 1 January 2005 and 31 December 2020, during which 50,557 births were registered. The study was approved by the Regional Committee on Research Ethics of Semmelweis University (approval number: 231/2017), and informed consent was obtained in accordance with institutional protocols.

### 2.2. Participants

**Figure 1:**
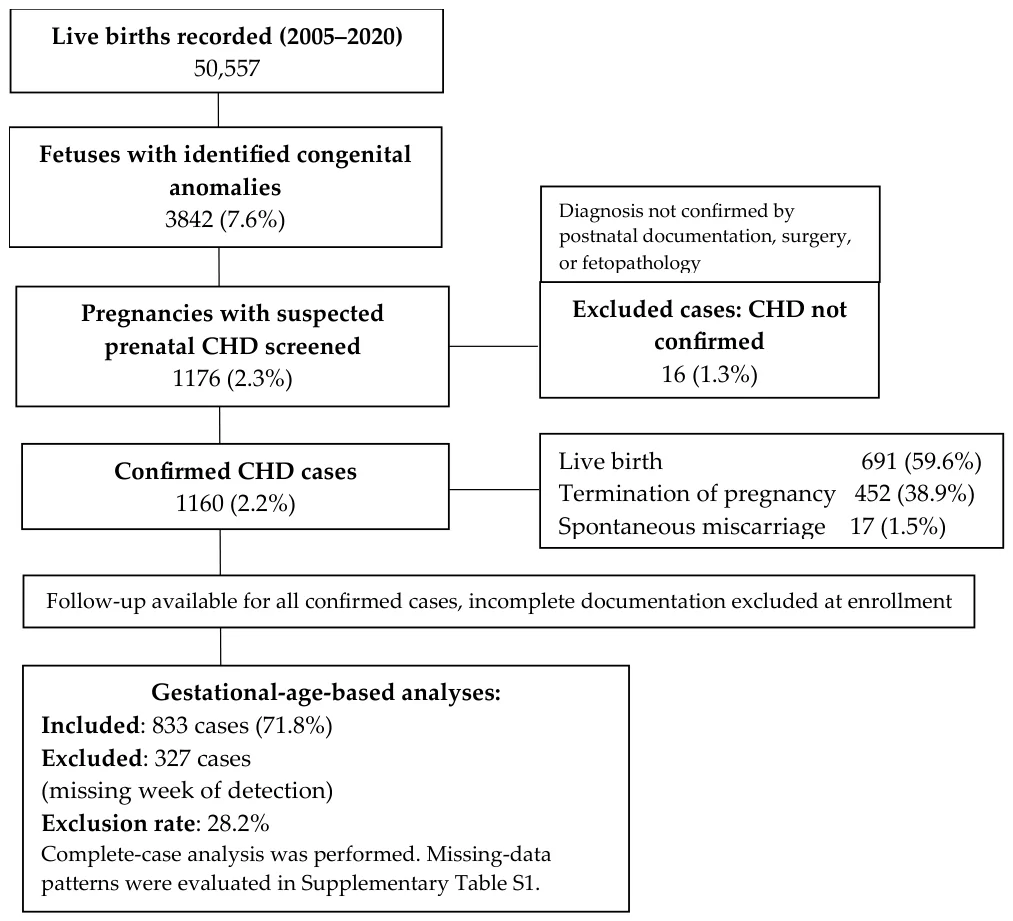
Flow diagram of congenital heart defect case identification and inclusion in the gestational-age-based analysis.

Eligible cases were pregnancies with prenatal ultrasound suspicion of CHD confirmed by postnatal echocardiography, surgical findings, or fetopathological examination. Cases were excluded if: (1) cardiac malformation could not be confirmed postnatally; (2) gestational age at detection was undocumented; or (3) documentation was insufficient for variable extraction. Of 1176 registry entries, 16 were excluded for unconfirmed CHD and 327 lacked documented timing of detection, yielding 833 cases for analysis. A participant flow diagram is provided in Figure 1 [12].

### 2.3. Data Sources and Measurements

Data were abstracted from the MedSol electronic medical record system, prenatal ultrasound reports, genetic counselling documentation, neonatal discharge summaries, and fetopathological reports. Variables were extracted using a standardized form and linked by coded identifiers. Ultrasound examinations were performed in the Ultrasound Laboratory of the Semmelweis University Obstetrics and Gynecology Clinic, Baross Street Department, using Medison Sonoace X8 (Medison Co., Ltd., Seoul, Republic of Korea), Samsung Medison UGEO H60 (Samsung Medison Co., Ltd., Seoul, Republic of Korea), Samsung Medison WS80A (Samsung Medison Co., Ltd.), and Philips^®^ HD 11XE (Philips Ultrasound, Amsterdam, The Netherlands) ultrasound machines, following American Heart Association fetal cardiac screening protocols including four-chamber and outflow-tract views [3,13]. Fetal echocardiography was performed by experienced fetal cardiologists when abnormalities were suspected [14].

### 2.4. Variables and Outcome Assessment

The primary outcome was the gestational week of prenatal diagnosis, defined as the completed gestational week at which a fetal cardiac abnormality was first documented in the prenatal record by ultrasound or fetal echocardiography. This definition included both complete and partial prenatal cardiac diagnoses. Diagnostic completeness was treated as a separate descriptive variable and was not considered part of the primary outcome definition.

Predictor variables included: maternal age (continuous, years; advanced maternal age defined as ≥35 years for descriptive purposes), twin pregnancy, oligohydramnios (defined as an amniotic fluid index (AFI) of ≤5 cm or a single deepest vertical pocket of <2 cm) polyhydramnios (defined as a deepest vertical pocket of ≥8 cm or an amniotic fluid index (AFI) of ≥24 cm), any chromosomal abnormality, Down syndrome specifically, and CHD severity [15,16].

CHD severity was classified as non-severe (ventricular septal defect, bicuspid aortic valve, mild pulmonary stenosis, mild coarctation) or severe (tetralogy of Fallot, transposition of the great arteries, hypoplastic left heart syndrome, truncus arteriosus, pulmonary atresia, total anomalous pulmonary venous return, critical coarctation, Ebstein anomaly) based on hemodynamic impact and neonatal intervention urgency, adapted from American Heart Association guidance, as can be seen in Table 1 [3].

This was assigned according to the final confirmed diagnosis rather than the initial prenatal impression. In cases with multiple cardiac lesions, classification was based on the lesion with the greatest expected haemodynamic impact and urgency of neonatal intervention. For lesions with broad severity spectra, such as coarctation of the aorta, pulmonary stenosis, and septal defects, severity classification was based on documented anatomical and haemodynamic features when available.

Prenatal diagnostic timing is likely influenced not only by haemodynamic severity but also by lesion-specific sonographic visibility. Defects that alter the four-chamber view or outflow-tract anatomy may be recognized earlier than lesions with subtler or evolving prenatal features. For example, ventricular septal defects and tetralogy of Fallot may be suspected when abnormalities are visible on standard four-chamber or outflow-tract views, whereas other lesions may be more dependent on image quality, gestational age, fetal position, or specialist assessment. Conversely, clinically important lesions such as coarctation of the aorta or transposition of the great arteries may vary substantially in prenatal detectability despite their clinical relevance.

Atrial septal defects were not analyzed as a separate prenatal diagnostic category because atrial-level communication is difficult to classify reliably in fetal life and the retrospective dataset did not consistently distinguish ostium primum, sinus venosus/caval-type, and secundum-type atrial communications. When atrial-level communication occurred as part of multiple CHDs, the case was retained and classified according to the principal lesion with the greatest expected haemodynamic impact or neonatal intervention relevance.

Amniotic fluid volume was assessed qualitatively during ultrasound and classified as normal, oligohydramnios, or polyhydramnios. Chromosomal abnormalities were confirmed by karyotyping or chromosomal microarray.

### 2.5. Statistical Methods

The gestational week of detection was analyzed as a continuous dependent variable. Distributional assumptions were assessed graphically using histograms and quantile–quantile plots and formally with the Shapiro–Wilk test; homogeneity of variances was evaluated with Levene’s test. In univariable analyses, associations between binary predictors and recognition timing were examined using Student’s *t*-test when normality and homogeneity of variance were met, Welch’s *t*-test when variances were unequal, and the Mann–Whitney U test with permutation-based confirmation (10,000 permutations) for non-normal or small-group comparisons [17,18]. Mean and median differences between groups were reported [19].

Multivariable linear regression with heteroscedasticity-robust standard errors (HC3) was used to evaluate variables associated with the gestational week of diagnosis. Candidate variables were selected based on clinical relevance and biological plausibility and included maternal age, twin pregnancy, amniotic fluid abnormalities, chromosomal abnormalities, Down syndrome, and selected cardiac diagnoses. Given the long study period, the calendar year of diagnosis was additionally considered to account for temporal changes in referral practice, screening policy, ultrasound technology, and fetal cardiology services. The primary fully adjusted model was used as the primary analysis; backward elimination, if performed, was considered exploratory. Multicollinearity was assessed using variance inflation factors (VIF > 5 indicating concern), and residual diagnostic plots were reviewed. Regression coefficients with 95% confidence intervals are reported. Analyses were performed in Python 3.9 using pandas, scipy, and statsmodels [19].

Diagnostic completeness was not included as a predictor in the primary multivariable model because it may occur downstream of diagnostic timing and could introduce circularity. Instead, recognition completeness was evaluated descriptively.

### 2.6. Bias and Missing Data

Referral bias was acknowledged; the tertiary centre cohort is enriched for severe anomalies. Verification bias was minimized by requiring confirmation of all diagnoses through independent postnatal evaluation, surgical findings, or fetopathological examination. Information bias was reduced through standardized clinical documentation and structured data extraction. Selection bias was minimized by analyzing consecutively recorded registry cases within a defined time period.

Missing gestational-week documentation was evaluated using Firth-penalized logistic regression because ordinary logistic regression showed sparse-data instability and complete or quasi-complete separation for rare lesion-specific variables. The dependent variable was exclusion from the gestational-age analytic cohort because the gestational week of prenatal diagnosis was unavailable. Candidate predictors included maternal age, twin pregnancy, amniotic fluid abnormalities, chromosomal status, predefined calendar period, and selected lesion-specific diagnoses. Calendar period was modelled categorically as 2005–2010, 2011–2015, and 2016–2020, with 2005–2010 as the reference period. Chromosomal status was modelled hierarchically as no chromosomal abnormality, Down syndrome, or other chromosomal abnormality. Penalized odds ratios with profile-likelihood 95% confidence intervals and profile-penalized likelihood-ratio *p* values are reported in Supplementary Table S1.

## 3. Results

### 3.1. Participants

Of 1176 registry entries initially identified, 833 pregnancies met inclusion criteria and comprised the analytic cohort (Figure 1). Sixteen cases were excluded for unconfirmed CHD, and 327 lacked documented gestational age at detection. Of 1176 registry entries initially identified, 16 were excluded because postnatal echocardiography, surgical findings, or fetopathological examination could not confirm CHD. The annual distribution shown in Table 2 therefore refers to the 1160 confirmed CHD cases, irrespective of whether the gestational week of prenatal diagnosis was documented. For each year, Table 2 reports the annual denominator, the number of cases with complete or partial prenatal recognition, and the corresponding annual recognition rate. The 327 confirmed cases without a documented gestational week of diagnosis were excluded from the gestational-age analytic cohort. Still, they were retained in this descriptive annual recognition table when recognition status was available.

Complete recognition indicated that the prenatal anatomical diagnosis matched the final confirmed cardiac diagnosis. Partial recognition indicated that CHD was identified before birth, but the anatomical diagnosis was incomplete or only partly consistent with the final diagnosis. Prenatal suspicion without the correct anatomical diagnosis indicated that a fetal cardiac abnormality was suspected prenatally and a gestational week of first suspicion was documented, but the final anatomical CHD diagnosis was not correctly established until postnatal, surgical, or fetopathological confirmation.

### 3.2. Descriptive Data

Baseline characteristics of the study population are presented in Figure 1. The median gestational age at detection was 21 weeks (interquartile range, 20–27 weeks), as shown in Table 3. Advanced maternal age (≥35 years) was present in 267 cases (32.1%); the median age was 31 years. Twin pregnancies accounted for 50 cases (6.0%), as shown in Table 4. Oligohydramnios was documented in 51 cases (6.1%) and polyhydramnios in 125 cases (15.0%). Chromosomal abnormalities were confirmed in 146 cases (17.5%), of which 75 (9.0%) were Down syndrome. Severe CHD was present in 643 cases (77.2%), as expressed in Table 4.

Diagnostic completeness was evaluated descriptively and was not included as an explanatory variable in the multivariable model, because it reflects the diagnostic process itself rather than a baseline maternal, fetal, or lesion-specific characteristic. Among the 833 cases included in the gestational-age-based analysis, complete prenatal recognition of the final cardiac diagnosis was achieved in 653 cases (78.4%). Partial recognition was present in 160 cases (19.2%). In 20 cases (2.4%), there was prenatal suspicion of a fetal cardiac abnormality, but the correct final anatomical diagnosis was not established until postnatal, surgical, or fetopathological confirmation.

Among the 833 cases included in the gestational-age analytic cohort, complete prenatal recognition of the final cardiac diagnosis was achieved in 653 cases (78.4%), and partial recognition was present in 160 cases (19.2%). Therefore, complete or partial prenatal recognition was achieved in 813 of 833 analytic cases (97.6%). In contrast, when all 1160 confirmed CHD cases were considered, complete or partial prenatal recognition was present in 911 cases (78.5%). This difference indicates that restricting to cases with a documented gestational week at diagnosis enriched the analytic cohort for cases with complete or partial prenatal recognition. Accordingly, the 97.6% recognition rate should be interpreted as applying to the gestational-age analytic cohort only, not to the entire confirmed CHD source cohort.

This descriptive classification was used to characterize diagnostic completeness within the cohort, whereas the primary regression analysis focused on factors associated with the gestational week of prenatal diagnosis.

### 3.3. Univariable Analyses

**Figure 2:**
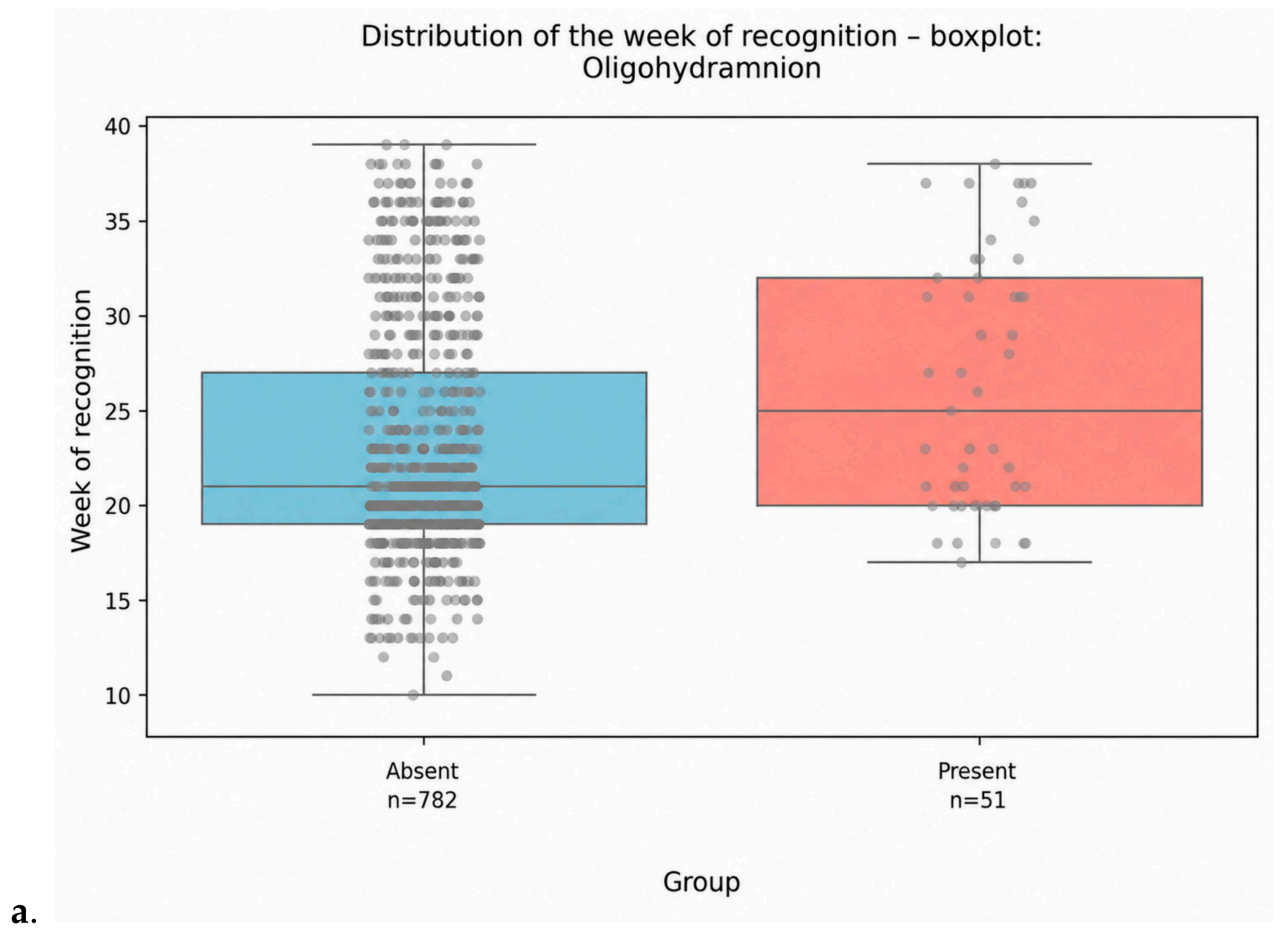

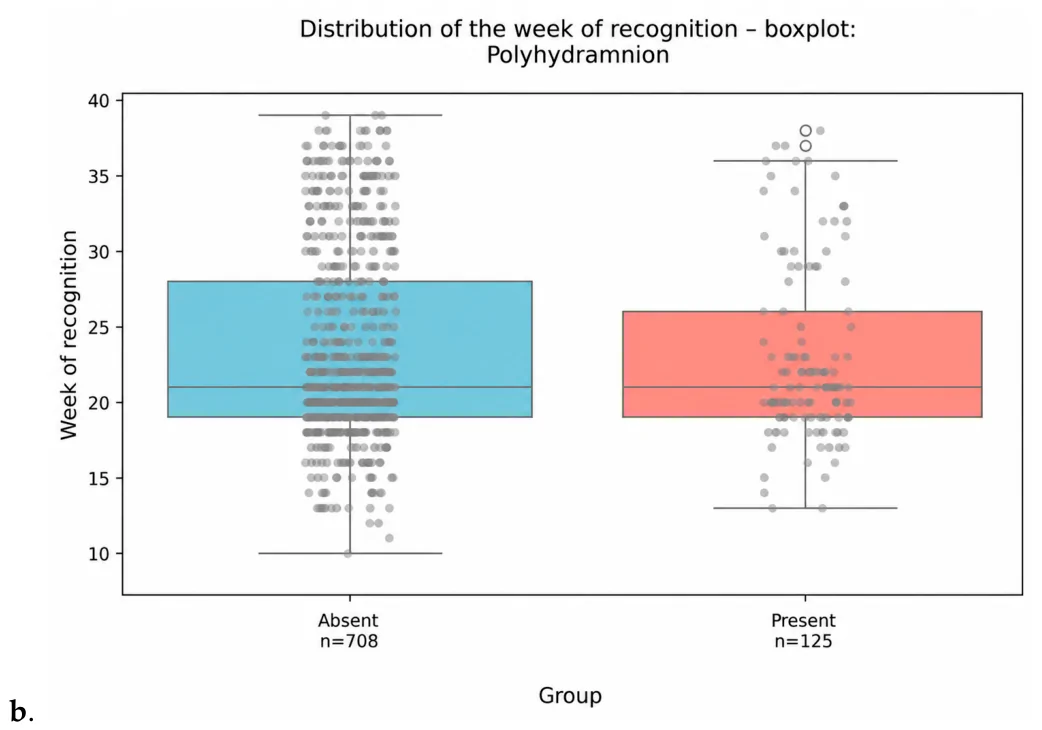
Gestational week of recognition stratified by (**a**,**b**) amniotic fluid abnormalities differentiating between significant and non-significant results of univariable analyses; for binary predictors, 0 indicates absence of the condition, and 1 indicates presence of the condition.

Associations between predictor variables and gestational age at detection are summarized in Table 4. Advanced maternal age was associated with significantly earlier detection (mean 21.7 vs. 24.1 weeks; mean difference −2.48 weeks; *p* < 0.001; 95% CI −3.3 to −1.6). Down syndrome was similarly associated with earlier recognition (mean 20.7 vs. 23.6 weeks; mean difference −2.85 weeks; *p* < 0.001, 95% CI −4.1 to −1.6). Chromosomal abnormalities as a broader category were associated with earlier detection in univariable analysis (mean difference −2.05 weeks; *p* = 0.0002, 95% CI −3.1 to −1.0). Among amniotic fluid abnormalities, oligohydramnios was associated with significantly later detection (mean 26.3 vs. 23.1 weeks; mean difference +3.17 weeks; *p* = 0.0018, 95% CI 1.2 to 5.1). In contrast, polyhydramnios showed no significant association (mean difference −0.48 weeks; *p* = 0.43, 95% CI −1.6 to 0.6), as can be seen in Figure 2. Twin pregnancy was also not significantly associated with gestational age at diagnosis (mean difference −1.40 weeks; *p* = 0.13, 95% CI −3.0 to 0.2).

The category of prenatal suspicion without correct anatomical diagnosis referred to cases in which a cardiac abnormality was suspected prenatally, but the final anatomical CHD diagnosis was not correctly established until postnatal, surgical, or fetopathological confirmation.

### 3.4. Multivariable Analyses

In the results of the primary fully adjusted multivariable linear regression model, calendar year was included as a continuous variable to account for temporal changes during the study period, and diagnostic-completeness variables were excluded from the predictor set. Chromosomal status was modelled hierarchically, with Down syndrome and other chromosomal abnormalities compared separately with cases without chromosomal abnormalities. The results of the multivariable linear regression analyses are shown in Table 5. 

The model was statistically significant and explained a limited proportion (12.2%) of the variability in the gestational week of prenatal diagnosis, with an adjusted R^2^ of 0.107, underscoring the multifactorial nature of diagnostic timing, which likely reflects not only the measured maternal, fetal, and lesion-specific variables, but also referral pathways, screening history, imaging conditions, and temporal changes in prenatal care, although the overall model was statistically significant (F-statistic = 5.246; *p* < 0.001). Robust HC3 standard errors were used to account for potential heteroscedasticity.

This low explanatory performance represents an important limitation and indicates that most variation in diagnostic timing was not captured by the available maternal, fetal, temporal, and lesion-specific variables. The model should therefore be interpreted as an adjusted association model rather than as a comprehensive predictive model of prenatal diagnostic timing.

Adjusted predictions were derived from the final multivariable linear regression model. Each plot shows the model-based predicted gestational week of prenatal diagnosis for the variable of interest while holding other covariates constant at the reference profile: continuous covariates at their sample mean, binary predictors as absent, and chromosomal status as no chromosomal abnormality. Lines represent adjusted mean predictions, with shaded areas or error bars indicating 95% confidence intervals.

**Figure 3:**
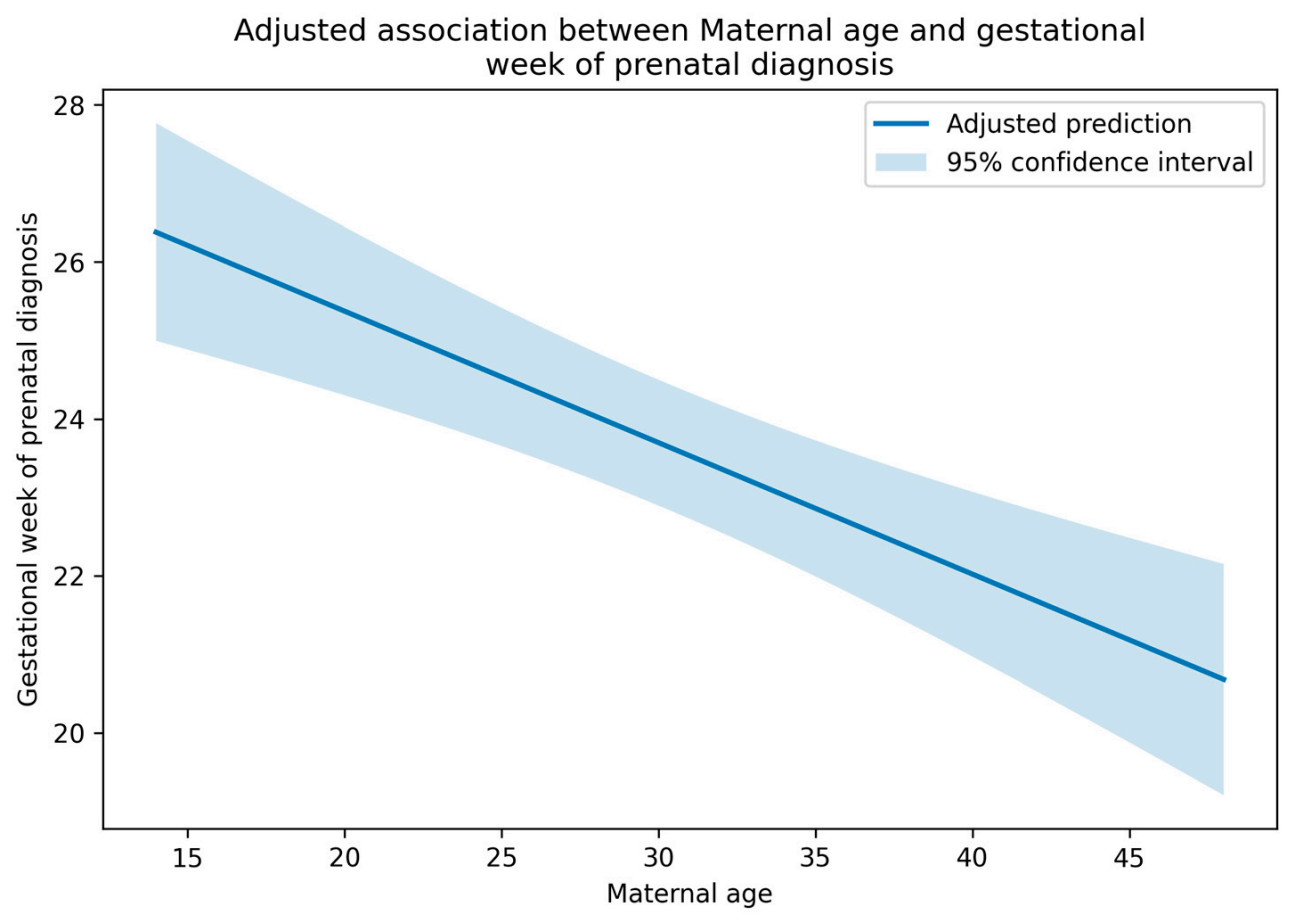
Relationship Between maternal age and gestational week of prenatal diagnosis after multivariable adjustment.

After multivariable adjustment, increasing maternal age was associated with earlier prenatal diagnosis, with each additional year of maternal age corresponding to a 0.17-week earlier diagnosis (β = −0.169, robust SE = 0.035, 95% CI −0.238 to −0.099; *p* < 0.001) as shown in Figure 3.

**Figure 4:**
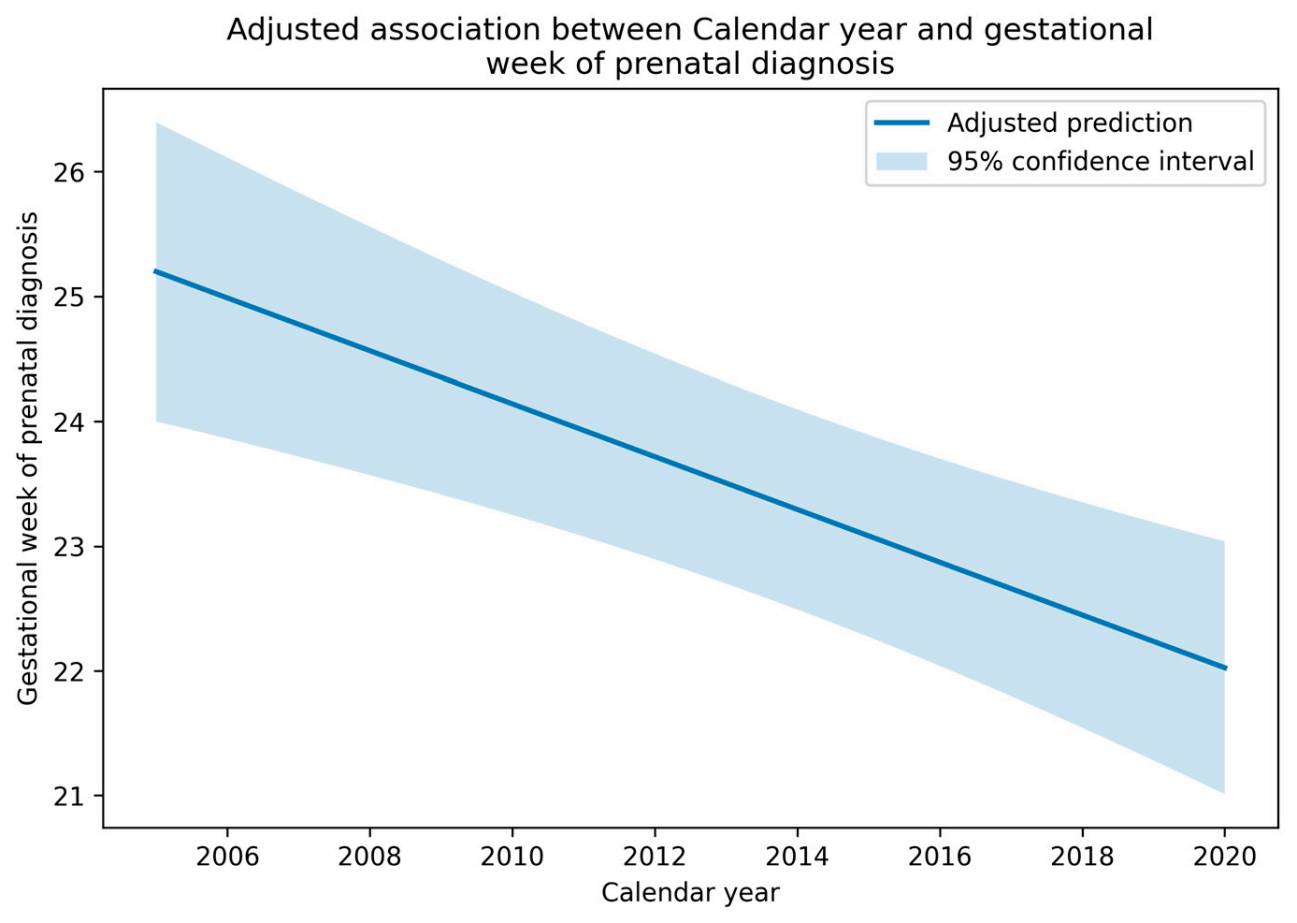
Relationship Between calendar year and gestational week of prenatal diagnosis after multivariable adjustment.

Calendar year was also independently associated with earlier diagnosis, with diagnoses occurring approximately 0.21 weeks earlier per calendar year after adjustment for the other variables (β = −0.211, robust SE = 0.051, 95% CI −0.311 to −0.111; *p* < 0.001) as shown in Figure 4. In contrast, coarctation of the aorta/aortic arch stenosis, pulmonary stenosis, and oligohydramnios were associated with later prenatal diagnosis. Coarctation of the aorta/aortic arch stenosis was associated with a 2.28-week later diagnosis (β = 2.276, robust SE = 0.758, 95% CI 0.787 to 3.764; *p* = 0.003), pulmonary stenosis with a 2.00-week later diagnosis (β = 1.996, robust SE = 0.825, 95% CI 0.376 to 3.617; *p* = 0.016), and oligohydramnios with a 2.10-week later diagnosis (β = 2.097, robust SE = 0.952, 95% CI 0.228 to 3.965; *p* = 0.028). 

Down syndrome showed a borderline association with earlier prenatal diagnosis, but this did not reach conventional statistical significance because the confidence interval crossed zero (β = −1.350, robust SE = 0.689, 95% CI −2.702 to 0.002; *p* = 0.050). Other variables, including ventricular septal defect, twin pregnancy, tetralogy of Fallot, transposition of the great arteries, atrioventricular septal defect, truncus arteriosus, aortic stenosis, polyhydramnios, Ebstein anomaly, double outlet right ventricle, hypoplastic left heart syndrome, univentricular heart, and other chromosomal abnormalities, were not independently associated with the gestational week of prenatal diagnosis in the adjusted model.

**Figure 5:**
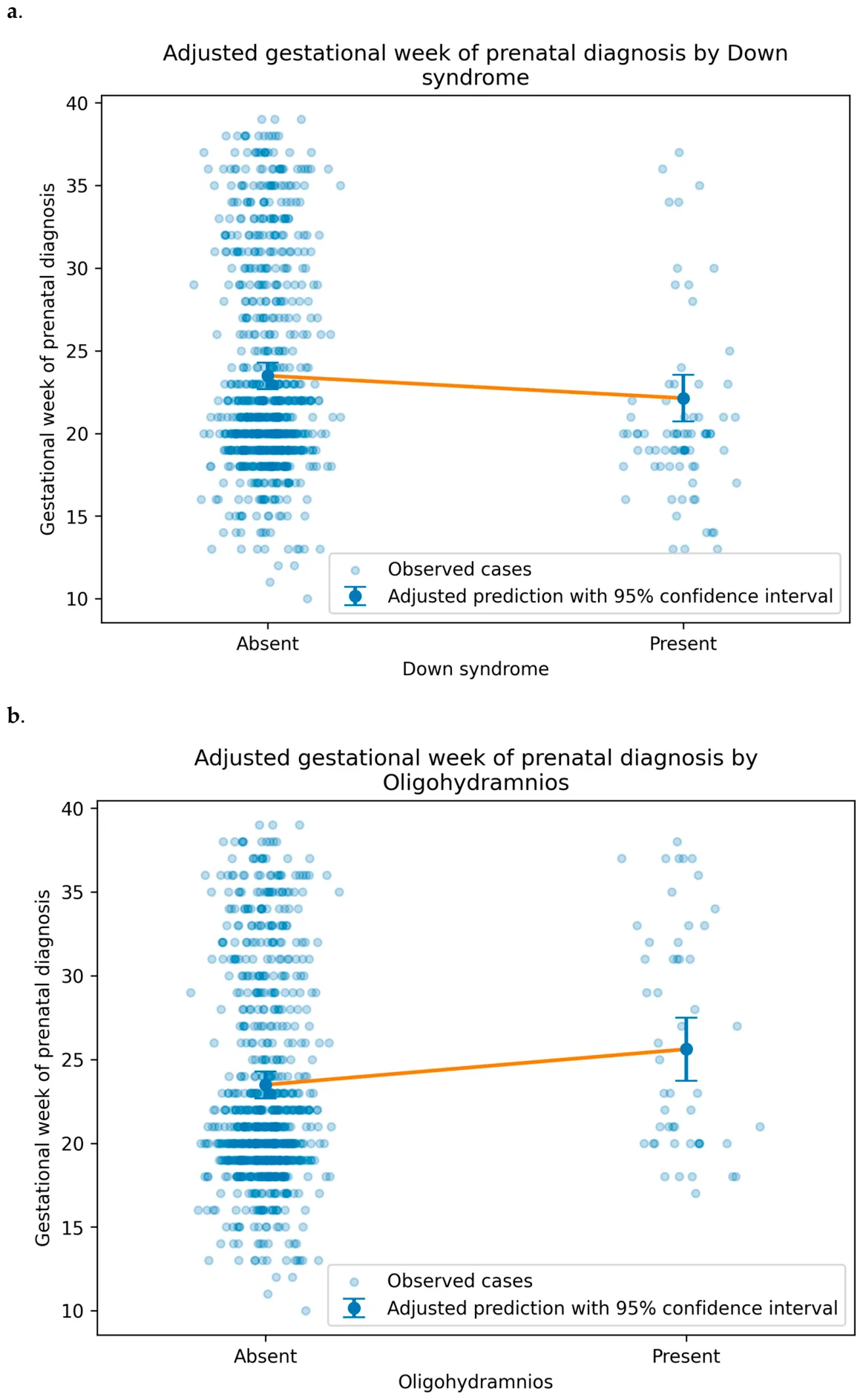

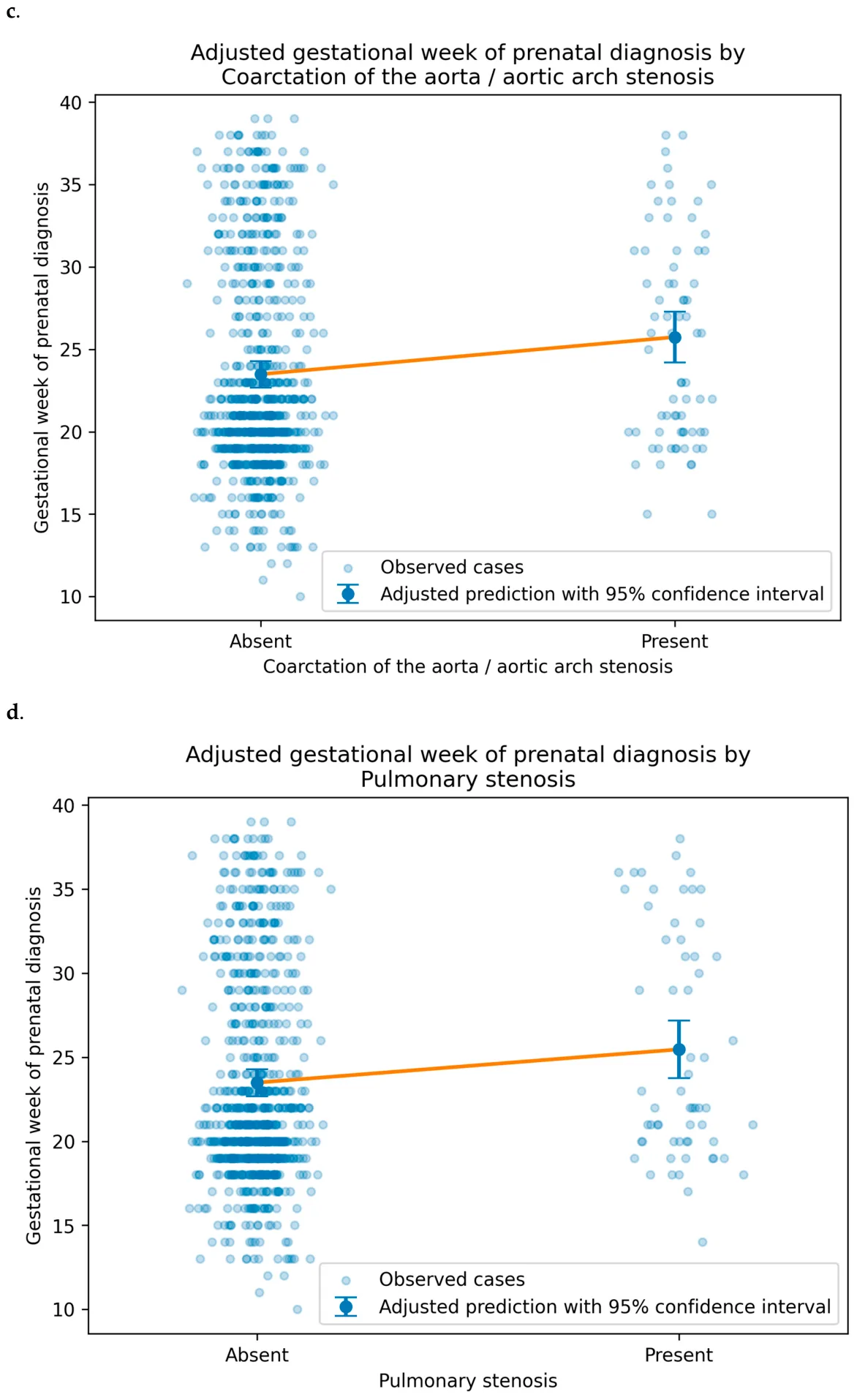
Multivariable-adjusted predicted gestational week of recognition by selected clinical (**a**,**b**) and cardiac (**c**,**d**) characteristics.

Adjusted model-based predictions for selected clinical and lesion-specific variables are shown in Figure 5. In each panel, pale blue dots represent observed cases, while the blue points with vertical error bars represent the model-based adjusted predicted gestational week of prenatal diagnosis with 95% confidence intervals. The orange line connects the adjusted predictions for the two categories and illustrates the direction of the adjusted association.

## 4. Discussion

### 4.1. Summary of Principal Findings

This study examined factors associated with the gestational week of diagnosis among pregnancies with prenatally recognized and subsequently confirmed congenital heart defect referred to a tertiary fetal cardiology centre. Therefore, the findings should be interpreted as associations within an already recognized referral cohort, rather than as determinants of prenatal CHD detection in the general pregnant population.

In this primary fully adjusted multivariable model, which included calendar year as a continuous variable and excluded diagnostic-completeness variables from the predictor set, several factors were associated with the gestational week of prenatal diagnosis.

Advancing maternal age was associated with earlier diagnosis, with each additional year of maternal age corresponding to diagnosis approximately 0.17 weeks earlier. Calendar year was also associated with earlier diagnosis, with diagnoses occurring approximately 0.21 weeks earlier per calendar year after adjustment for other variables. In contrast, coarctation of the aorta/aortic arch stenosis, pulmonary stenosis, and oligohydramnios were associated with later prenatal diagnosis, corresponding to delays of approximately 2.28, 2.00, and 2.10 weeks, respectively. Down syndrome showed a borderline association with earlier diagnosis; however, this did not reach conventional statistical significance because the confidence interval crossed zero.

Several variables that appeared clinically relevant or were significant in earlier analyses did not retain statistically significant associations in the revised adjusted model, including ventricular septal defect, tetralogy of Fallot, twin pregnancy, transposition of the great arteries, atrioventricular septal defect, truncus arteriosus, aortic stenosis, polyhydramnios, hypoplastic left heart syndrome, univentricular heart, and other chromosomal abnormalities. These findings highlight the importance of accounting for correlated maternal, fetal, temporal, and lesion-specific factors when evaluating diagnostic timing in prenatal CHD cohorts.

The explanatory performance of the adjusted model was limited and represents a major limitation of the study. The adjusted model was statistically significant but explained a limited proportion of variability in the gestational week of diagnosis, underscoring the multifactorial nature of diagnosis among the recognition of prenatally referred CHD cases. Diagnostic timing is likely influenced by additional referral-, imaging-, and care pathway-related factors not fully captured in the present dataset. Further prospective studies are planned, together with retrospective collection of these additional variables where available, to better characterize determinants of diagnostic timing.

### 4.2. Comparison with the Existing Literature

The association between advanced maternal age and earlier detection is consistent with established prenatal care pathways. Women of advanced maternal age are more likely to undergo enhanced surveillance, including first-trimester screening with nuchal translucency measurement and cell-free DNA testing, increasing opportunities for early referral to fetal echocardiography [3,16,20]. Recommendations for detailed fetal anatomic ultrasound examination in pregnant individuals aged 35 years and older may further support earlier identification of cardiac anomalies [21,22]. The higher risk of aneuploidy with advancing maternal age also prompts more intensive genetic screening and earlier fetal cardiac evaluation when abnormalities are suspected [16,21,23,24].

The observed association between calendar year and earlier prenatal diagnosis is also clinically plausible. The study period spanned 2005 to 2020, during which prenatal screening policies, ultrasound technology, outflow-tract imaging, first-trimester screening, genetic testing, referral pathways, and fetal cardiology services changed substantially. The negative association between calendar year and the gestational week of diagnosis suggests that prenatal CHD diagnoses occurred progressively earlier over time within this referral cohort. However, this temporal association should not be interpreted as evidence of a uniform improvement across all diagnostic categories. Changes in screening practice and imaging quality may have affected some lesion types or genetically associated cases more than others.

The borderline tendency toward earlier detection in fetuses with Down syndrome is consistent with the high prevalence of congenital heart defects in this population, estimated at 40–50% [21,25]. First-trimester markers such as increased nuchal translucency may trigger early fetal echocardiography referral, and the American Heart Association notes that increased nuchal translucency accounts for many early cardiac diagnoses among first- and early second-trimester referrals [3,26,27,28,29,30,31]. Characteristic lesions, particularly atrioventricular septal defects, may also be readily identifiable on standard four-chamber views [32,33].

The associations observed for advanced maternal age and Down syndrome should be interpreted primarily in the context of established prenatal surveillance and referral pathways. These factors may not directly cause earlier cardiac recognition; rather, they are likely to increase the probability of earlier targeted screening, genetic evaluation, and referral for fetal echocardiography. Accordingly, they should be considered clinical markers of increased surveillance intensity rather than modifiable determinants of diagnostic timing.

Oligohydramnios was associated with a later gestational week of diagnosis; however, the temporal relationship cannot be entirely established from the available data. Reduced amniotic fluid may impair acoustic windows and limit fetal cardiac visualization, but oligohydramnios may also develop later in pregnancy or reflect underlying fetal, placental, or maternal conditions [34,35,36,37]. Therefore, this association should be interpreted cautiously and should not be considered evidence that oligohydramnios directly causes delayed diagnosis.

However, among the examined variables, oligohydramnios may have the most direct practical relevance for clinical workflow. Although the temporal relationship between oligohydramnios and CHD diagnosis cannot be established in this retrospective analysis, reduced amniotic fluid may impair acoustic windows and limit fetal cardiac visualization. Therefore, when oligohydramnios is present and cardiac views are suboptimal, earlier repeat imaging or referral for specialized fetal echocardiography may be appropriate rather than delayed follow-up [13].

The lesion-specific findings also require careful interpretation. Coarctation of the aorta/aortic arch stenosis and pulmonary stenosis were associated with later prenatal diagnosis in the adjusted model. This may reflect the diagnostic complexity of obstructive and outflow-tract lesions, some of which can evolve during gestation or may be difficult to distinguish earlier in pregnancy depending on fetal position, acoustic windows, and the degree of haemodynamic expression at the time of examination [38,39]. In contrast, ventricular septal defects and tetralogy of Fallot were not statistically significant after adjustment in the revised model, suggesting that their apparent associations in earlier analyses may have been influenced by correlated clinical or temporal factors [38].

### 4.3. Clinical Implications

These findings have primarily interpretive rather than directly prescriptive clinical implications. They suggest that the gestational week of prenatal CHD diagnosis among referred and prenatally recognized cases is shaped by a combination of surveillance pathways, temporal changes in prenatal care, fetal conditions, and lesion-specific sonographic visibility. The associations with maternal age and calendar year support the relevance of screening pathways and evolving diagnostic practice, but they should not be interpreted as evidence that these factors directly determine diagnostic timing [21]. Among the examined variables, oligohydramnios may have the most direct practical relevance for clinical workflow. Although the temporal relationship between oligohydramnios and CHD diagnosis cannot be established in this retrospective analysis, reduced amniotic fluid may impair acoustic windows and limit fetal cardiac visualization. Therefore, when oligohydramnios is present and cardiac views are suboptimal, earlier repeat imaging or referral for specialized fetal echocardiography may be appropriate rather than delayed follow-up [34]. Therefore, the results should be viewed as hypothesis-generating and as a basis for future studies that incorporate referral timing, screening history, ultrasound quality, and calendar-period effects [39].

From a methodological perspective, these results reinforce the value of multivariable analytical approaches in prenatal diagnostic research. The discrepancy between univariable and multivariable results for several variables emphasizes that crude associations may be misleading when correlated factors are not considered simultaneously. Univariable analyses identified several apparently significant associations that were not confirmed as independent effects, which could lead to erroneous conclusions if multivariable modelling was not performed. This observation is consistent with broader principles of epidemiological research design and has implications for the interpretation of prior studies that relied solely on univariable comparisons.

### 4.4. Strengths and Limitations

This study has several strengths. The cohort was drawn from a national tertiary referral centre with standardized diagnostic protocols and comprehensive postnatal confirmation of all diagnoses. The 15-year study period provided a substantial sample size and enabled the simultaneous examination of multiple maternal and fetal factors. The statistical approach used a fully adjusted multivariable model, heteroscedasticity-robust standard errors, hierarchical modelling of chromosomal status, and calendar-year adjustment, thereby addressing several potential sources of bias.

Several limitations warrant consideration. First, as a single-centre study conducted at a tertiary referral centre, the cohort is enriched for severe and complex anomalies, and milder lesions managed outside the referral pathway may be underrepresented [40]. This referral bias may limit generalisability to lower-risk populations or community-based settings. Second, the gestational age at prenatal diagnosis was unavailable for 28.2% of cases, which may introduce selection bias despite the absence of an apparent association with calendar year or diagnostic completeness. Third, important determinants of diagnostic timing, including referral indication, timing of the first abnormal ultrasound finding, gestational age at first screening examination, operator experience, fetal position, maternal body habitus, image quality, socioeconomic status, and regional access to prenatal care, were not available in the dataset [7,41,42].

Temporal change remains an additional limitation, although calendar year was included in the model. Prenatal screening policies, ultrasound technology, referral pathways, first-trimester screening, and fetal cardiology services changed substantially between 2005 and 2020. These changes may not have affected all diagnostic groups equally. For example, improvements in outflow-tract imaging or changes in aneuploidy screening may have preferentially influenced the recognition of specific lesions or genetically associated cases. Therefore, residual temporal confounding cannot be excluded [43].

A major limitation is the limited explanatory performance of the fully adjusted model. Although statistically significant, the model explained only a small proportion of the variability in the gestational week of prenatal diagnosis, with an adjusted R^2^ of 0.107. This indicates that most variation in the gestational week of prenatal diagnosis was explained by factors not captured in the available dataset. The model should therefore be interpreted as identifying associations within the available referral cohort rather than as a comprehensive predictive model of prenatal diagnostic timing. Future studies should incorporate detailed referral pathways, screening history, ultrasound quality, fetal position, operator experience, and lesion-specific imaging characteristics to characterize determinants of diagnostic timing better.

In the Firth penalized missing-data model, missing gestational-week documentation was associated with several observed clinical, temporal, chromosomal, and lesion-specific variables, including twin pregnancy, polyhydramnios, ventricular septal defect, tetralogy of Fallot, truncus arteriosus, hypoplastic left heart syndrome, pulmonary atresia, mitral stenosis, other chromosomal abnormalities, and calendar period 2016–2020. These findings indicate that missing diagnostic-timing data were not completely random. Therefore, the primary results should be interpreted as complete-case associations, and selection bias related to the availability of the documented gestational week cannot be excluded. Estimates for rare lesion categories, particularly mitral stenosis, should be interpreted cautiously because of sparse data.

The higher complete-or-partial recognition rate in the gestational-age analytic cohort compared with the full confirmed CHD cohort suggests that complete-case selection may have preferentially retained cases with better documented or more complete prenatal diagnostic information.

## 5. Conclusions

In this tertiary referral-centre cohort of pregnancies with prenatally recognized and confirmed congenital heart defect, the gestational week of prenatal diagnosis was associated with maternal age, calendar year, oligohydramnios, and selected lesion-specific diagnoses. Increasing maternal age and later calendar year were associated with earlier diagnosis, whereas oligohydramnios, coarctation of the aorta/aortic arch stenosis, and pulmonary stenosis were associated with later diagnosis. Down syndrome showed only a borderline association with earlier diagnosis in the revised adjusted model. Several variables that appeared relevant in univariable analyses did not retain statistically significant associations after multivariable adjustment, highlighting the importance of accounting for correlated maternal, fetal, temporal, and lesion-specific factors. Because the cohort included only prenatally recognized and referred cases and the model explained a limited proportion of variation in diagnostic timing, these findings should be interpreted as observational associations within a referral cohort rather than determinants of prenatal CHD detection in the general population. Future studies should incorporate referral pathways, screening history, ultrasound quality, operator experience, fetal position, and lesion-specific imaging characteristics to better characterize factors influencing the timing of prenatal CHD diagnosis.

## Figures and Tables

**Table 1:** Classification of congenital heart defects into severe and non-severe categories.

Congenital Heart Defect (CHD)	Non-Severe	Severe
Septal Defects	Ventricular Septal Defect (VSD)	Tetralogy of Fallot (TOF)
		Atrioventricular Septal Defect (AVSD)
		Double Outlet Right Ventricle (DORV)
		Truncus Arteriosus
Great Vessel and Circulatory Abnormalities	Coarctation of the Aorta	Interrupted Aortic Arch (IAA)
		Transposition of the Great Arteries (TGA)
		Hypoplastic Left Heart Syndrome (HLHS)
	Partial Anomalous Venous Connection (PAPVC)	Total Anomalous Pulmonary Venous Return (TAPVR)
Valve and Outflow Tract Defects	Bicuspid Aortic Valve	Pulmonary Atresia
	Pulmonary Stenosis	Ebstein Anomaly
	Mitral Valve Stenosis	Critical Aortic Stenosis

**Table 2:** Annual number of confirmed CHD cases and complete or partial prenatal recognition, 2005–2020.

Year	Number of Total Cases (n)	Number of Cases with Complete or Partial Recognition (n)	Rate of Complete or Partial Recognition (%)
2005	49	39	79.6%
2006	46	40	87.0%
2007	52	40	76.9%
2008	66	55	83.3%
2009	56	44	78.6%
2010	57	41	71.9%
2011	74	62	83.8%
2012	94	75	79.8%
2013	92	67	72.8%
2014	75	60	80.0%
2015	93	63	67.7%
2016	72	44	61.1%
2017	94	69	73.4%
2018	83	72	86.7%
2019	71	66	93.0%
2020	86	74	86.0%

**Table 3:** Distribution of gestational week of prenatal diagnosis in the gestational-age analytic cohort.

Gestational Week of Prenatal Diagnosis (Weeks)	Number of Cases (n)	Gestational Week of Prenatal Diagnosis (Weeks)	Number of Cases (n)
10	1	26	16
11	0	27	17
12	2	28	17
13	12	29	21
14	10	30	19
15	13	31	23
16	22	32	21
17	23	33	20
18	66	34	18
19	105	35	22
20	118	36	22
21	76	37	15
22	64	38	11
23	43	39	3
24	19	40	0
25	14		
Total:	833		
Median:	21		

**Table 4:** Univariable comparisons of gestational week of prenatal diagnosis by maternal, fetal, and congenital heart defect characteristics.

Variable	Absent, n	Present, n	Avg. Absent (Weeks)	Avg. Present (Weeks)	CI95 Low (%)	CI95 High (%)	Median Present (Weeks)	Chosen Test	*p* Value
Advanced maternal age (>34)	566	267	24.1	21.7	−3.3	−1.6	20	Welch *t*-test	<0.001
Down syndrome	758	75	23.6	20.7	−4.1	−1.6	20	Welch *t*-test	<0.001
Chromosomal abnormalities	687	146	23.7	21.7	−3.1	−1	20	Welch *t*-test	<0.001
Oligohydramnios	782	51	23.1	26.3	1.2	5.1	25	Welch *t*-test	0.002
Coarctation of the aorta/Aortic arch stenosis	761	72	23.1	25.5	0.8	3.8	25.5	Student *t*-test	0.003
Pulmonary stenosis	772	61	23.2	25.4	0.5	4	22	Student *t*-test	0.008
Dilatative Cardiomyopathy (DCM)	821	12	23.4	19.4	−5.6	−2.4	20	Mann–Whitney U test	0.024
Ventricular septal defect (VSD)	515	318	23.7	22.8	−1.8	−0.1	21	Welch *t*-test	0.033
Transposition of the great arteries (TGA)	778	55	23.2	24.8	−0.3	3.4	22	Student *t*-test	0.079
tetralogy of Fallot	765	68	23.4	22.3	−2.5	0.2	21	Welch *t*-test	0.088
Atrioventricular septal defect (AVSD)	744	89	23.4	22.5	−2.2	0.3	20	Welch *t*-test	0.121
Twin pregnancy	783	50	23.4	22	−3	0.2	20	Student *t*-test	0.127
Polyhydramnios	708	125	23.4	22.9	−1.6	0.6	21	Student *t*-test	0.429

**Table 5:** Multivariable linear regression analysis of the gestational week of recognition.

Variable	Coefficient	Robust SE	t Value	*p* Value	95% CI Low	95% CI High
Constant	28.756	1.143	25.169	<0.001	26.513	30.999
Maternal age	−0.169	0.035	−4.781	<0.001	−0.238	−0.099
Calendar year (centred)	−0.211	0.051	−4.126	<0.001	−0.311	−0.111
Coarctation of the aorta	2.276	0.758	3.000	0.003	0.787	3.764
Pulmonary stenosis	1.996	0.825	2.418	0.016	0.376	3.617
Oligohydramnios	2.097	0.952	2.203	0.028	0.228	3.965
Down syndrome	−1.35	0.689	−1.959	0.050	−2.702	0.002
Ventricular septal defect	−0.758	0.465	−1.632	0.103	−1.67	0.153
Twin pregnancy	−1.188	0.787	−1.511	0.131	−2.732	0.356
tetralogy of Fallot	−0.943	0.662	−1.424	0.155	−2.243	0.357
Transposition of the great arteries	1.161	0.954	1.217	0.224	−0.712	3.034
Atrioventricular septal defect	−0.794	0.661	−1.201	0.230	−2.091	0.503
Truncus arteriosus	−1.469	1.303	−1.128	0.260	−4.026	1.088
Aortic stenosis	1.059	1.079	0.982	0.327	−1.059	3.177
Polyhydramnios	−0.593	0.627	−0.946	0.344	−1.823	0.637
Ebstein’s anomaly	1.162	3.114	0.373	0.709	−4.951	7.276
Double outlet right ventricle	0.499	1.394	0.358	0.720	−2.236	3.235
Hypoplastic left heart syndrome	0.179	0.755	0.237	0.813	−1.303	1.66
Univentricular heart	−0.178	1.278	−0.139	0.889	−2.688	2.331
Other chromosomal abnormalities	0.095	0.743	0.128	0.899	−1.364	1.554

## Data Availability

The data presented in this study are available on request from the corresponding author. The data are not publicly available due to privacy and ethical reasons.

## Supplementary Materials

The following supporting information can be downloaded at: https://www.mdpi.com/article/10.3390/children13081090/s1, Table S1: Firth Penalised Logistic Regression Analysis of Factors Associated with Missing Gestational-Week Documentation.

## Abbreviations

The following abbreviations are used in this manuscript:

Abbreviation	Meaning
AFI	Amniotic Fluid Index
Avg	Average
AVSD	Atrioventricular Septal Defect
CHD	Congenital Heart Defects
CI	Confidence Interval
CI95	95% Confidence Interval
Co. LTD.	Company, Limited
CoA	Coarctation of the Aorta
DCM	Dilatative Cardiomyopathy
DORV	Double Outlet Right Ventricle
DVP	Deepest Vertical Pocket
HC	Heteroscedasticity
HLHS	Hypoplastic Left Heart Syndrome
IAA	Interrupted Aortic Arch
PAPVC	Partial Anomalous Venous Connection
TAPVR	Total Anomalous Pulmonary Venous Return
TGA	Transposition of the Great Arteries
ToF	Tetralogy of Fallot
VIF	Variance inflation factors
VSD	Ventricular Septal Defect
