# Supplementary material for: Factors Associated with Gestational Week of Diagnosis Among Pregnancies with Prenatally Recognized Congenital Heart Disease: A Tertiary Referral-Centre Cohort Study
Source: Children (Basel). 2026 Aug 17;13(8):1090. doi: 10.3390/children13081090 (PMC13510979; doi:10.3390/children13081090)
Supplement: Supplementary file 1 [file children-13-01090-s001.zip › children-4426139-supplementary.pdf]

## Supplementary Table

**Supplementary Table S1.** Firth Penalised Logistic Regression Analysis of Factors Associated With Missing Gestational-Week Documentation

| Variable | Penalised log-odds coefficient | Standard error | Penalised odds ratio | 95% CI for odds ratio | p value |
|---|---|---|---|---|---|
| Polyhydramnios | -1.815 | 0.443 | 0.163 | 0.060-0.360 | <0.001 |
| Twin pregnancy | 0.977 | 0.254 | 2.656 | 1.612-4.340 | <0.001 |
| Hypoplastic left heart syndrome (HLHS) | -1.343 | 0.417 | 0.261 | 0.103-0.557 | <0.001 |
| Constant | -1.603 | 0.446 | 0.201 | 0.090-0.479 | <0.001 |
| Other chromosomal abnormality | -1.286 | 0.468 | 0.276 | 0.098-0.641 | 0.002 |
| Pulmonary atresia | -0.925 | 0.376 | 0.397 | 0.176-0.791 | 0.007 |
| Truncus arteriosus | -1.762 | 0.851 | 0.172 | 0.019-0.675 | 0.008 |
| Calendar period 2016-2020 | -0.518 | 0.206 | 0.596 | 0.397-0.892 | 0.008 |
| Tetralogy of Fallot | -0.883 | 0.408 | 0.413 | 0.172-0.868 | 0.017 |
| Mitral stenosis | -2.384 | 1.510 | 0.092 | 0.001-0.771 | 0.022 |
| Ventricular septal defect (VSD) | 0.351 | 0.176 | 1.421 | 1.015-1.989 | 0.036 |
| Univentricular heart | -0.918 | 0.585 | 0.399 | 0.105-1.107 | 0.080 |
| Maternal age | 0.019 | 0.014 | 1.019 | 0.992-1.047 | 0.163 |
| Oligohydramnios | 0.342 | 0.301 | 1.407 | 0.769-2.492 | 0.261 |
| Down syndrome | -0.309 | 0.303 | 0.734 | 0.401-1.305 | 0.299 |
| Double outlet right ventricle (DORV) | -0.480 | 0.611 | 0.619 | 0.159-1.813 | 0.405 |
| Transposition of the great arteries (TGA) | -0.267 | 0.332 | 0.766 | 0.388-1.429 | 0.412 |
| Pulmonary stenosis | 0.225 | 0.286 | 1.252 | 0.702-2.159 | 0.436 |
| Interrupted aortic arch (IAA) | -0.640 | 0.949 | 0.527 | 0.055-2.481 | 0.453 |
| Ebstein anomaly | 0.480 | 0.813 | 1.616 | 0.287-6.725 | 0.546 |
| Coarctation of the aorta | 0.142 | 0.281 | 1.152 | 0.653-1.966 | 0.616 |
| Calendar period 2011-2015 | -0.077 | 0.202 | 0.926 | 0.624-1.376 | 0.703 |
| Atrioventricular septal defect (AVSD) | -0.026 | 0.299 | 0.974 | 0.533-1.725 | 0.931 |
| Aortic stenosis | -0.024 | 0.457 | 0.976 | 0.368-2.252 | 0.958 |

**Footnote for Table S1.** The dependent variable was missing gestational-week documentation, defined as exclusion from the gestational-age analytic cohort because gestational week of prenatal diagnosis was unavailable. Firth penalised logistic regression was used because ordinary logistic regression showed sparse-data instability and complete or quasi-complete separation for rare lesion-specific variables. Odds ratios greater than 1 indicate higher odds of missing gestational-week documentation, whereas odds ratios less than 1 indicate lower odds. Calendar period was included as a predefined categorical variable, with 2005-2010 as the reference period. Chromosomal status was modelled hierarchically, with no chromosomal abnormality as the reference category. Penalised odds ratios with profile-likelihood 95% confidence intervals and profile-penalised likelihood-ratio p-values are shown for predictors; the intercept is shown for completeness, and its p-value was calculated using a Wald test. Estimates for very rare lesion categories, particularly mitral stenosis, should be interpreted cautiously because of sparse data.
